# Assessment of Phasic Changes of Vascular Size by Automated Edge Tracking-State of the Art and Clinical Perspectives

**DOI:** 10.3389/fcvm.2021.775635

**Published:** 2022-01-21

**Authors:** Luca Mesin, Stefano Albani, Piero Policastro, Paolo Pasquero, Massimo Porta, Chiara Melchiorri, Gianluca Leonardi, Carlo Albera, Paolo Scacciatella, Pierpaolo Pellicori, Davide Stolfo, Andrea Grillo, Bruno Fabris, Roberto Bini, Alberto Giannoni, Antonio Pepe, Leonardo Ermini, Stefano Seddone, Gianfranco Sinagra, Francesco Antonini-Canterin, Silvestro Roatta

**Affiliations:** ^1^Mathematical Biology and Physiology, Department of Electronics and Telecommunications, Politecnico di Torino, Turin, Italy; ^2^SC Cardiologia Ospedale Regionale U. Parini, Aosta, Italy; ^3^Department of Medical, Surgical and Health Sciences, Universitá di Trieste, Trieste, Italy; ^4^Department of Medical Sciences, Universitá di Torino, Turin, Italy; ^5^Robertson Centre for Biostatistics, Research Institute of Health and Wellbeing, University of Glasgow, Glasgow, United Kingdom; ^6^Chirurgia Generale e Trauma Team GOM Niguarda, Milan, Italy; ^7^Scuola Superiore Sant'Anna, Pisa, Italy; ^8^Fondazione Toscana G. Monasterio, Pisa, Italy; ^9^Highly Specialized in Rehabilitation Hospital-ORAS S.p.A., Motta di Livenza, Italy; ^10^Ospedale Unico di Santorso, AULSS7 Pedemontana, Italy; ^11^Integrative Physiology Lab, Department of Neuroscience, Universitá di Torino, Turin, Italy

**Keywords:** inferior vena cava, arterial stiffness, ultrasound imaging, pulsatility, fluid volume assessment, right atrial pressure

## Abstract

Assessment of vascular size and of its phasic changes by ultrasound is important for the management of many clinical conditions. For example, a dilated and stiff inferior vena cava reflects increased intravascular volume and identifies patients with heart failure at greater risk of an early death. However, lack of standardization and sub-optimal intra- and inter- operator reproducibility limit the use of these techniques. To overcome these limitations, we developed two image-processing algorithms that quantify phasic vascular deformation by tracking wall movements, either in long or in short axis. Prospective studies will verify the clinical applicability and utility of these methods in different settings, vessels and medical conditions.

## 1. Introduction

Currently, the invasive measurement of central venous pressure (CVP) to estimate right atrial pressure (RAP) is routinely used only in critically ill patients to assess cardiac hemodynamics and volume status. Medical devices that provide this information non-invasively are under development, but not routinely used yet in clinical practice (1). A widely adopted non-invasive approach to estimate RAP is based on ultrasound (US) imaging of the inferior vena cava (IVC) diameter and of its respiratory changes (2, 3). These changes can be expressed in terms of the caval index (CI), defined as the variation of the vessel diameter during a respiration cycle, relative to the maximum diameter (4). This approach is not standardized (5) [for instance, it is performed in either long (6) or short axis (7) views], is operator-dependent (8) [with an important effect of experience (9)] and prone to measurement errors (10) [e.g., due to movements (8, 11) or to irregular shape of the IVC (12)]. Therefore, a single measurement might only provide limited—and misleading—information. The following sources of variability of the standard US approach have been investigated (8): different respiration cycles (coefficient of variation, CoV=15%), specific longitudinal section (CoV=40%), inter- and intra-operator variability (CoV 35 and 28%, respectively). Furthermore, recent studies indicated that IVC collapsibility assessed with current methods is not a reliable predictor of fluid responsiveness (13) and its correlation with RAP is only modest (14, 15).

Alternative approaches to assess, non-invasively, the CVP or volemic status have been proposed. For instance, there is a good correlation between CVP with pressure measured in superficial veins at the forearm with an US probe equipped with a pressure transducer (16). Moreover, measuring with US the ratio of the internal jugular vein during a Valsalva maneuver to that at rest identifies patients with heart failure with more severe intravascular congestion at greater risk of poorer outcomes (17, 18). However these techniques are not routinely used in clinical practice, as they require a more robust validation.

The assessment of arterial wall properties is also useful to characterize the health of the cardiovascular system and can improve prediction of cardiovascular events beyond conventional risk factors (19). Measurement of aortic pulse wave velocity (PWV) is considered the current gold standard to assess arterial stiffness. It has acceptable degree of accuracy and reliability (10) and has demonstrated to predict cardiovascular events for patients with different cardiovascular risk factors or diseases (20), but requires a specialized equipment (arterial tonometry, piezoelectric sensors, photoplethysmography) and additional time and resources. Moreover, current assessment of PWV may exclude the proximal segment of the aorta and might not identify pathological segments along the arterial tree, the deformation of which could, potentially, be assessed by US if a reliable method existed (21, 22).

Recently, we have started to address some of the above mentioned issues and developed two semi-automated methods to delineate and track displacements of the IVC borders in long (4, 8, 23, 24) or short axis views (12). Our approaches could reduce the inter/intra-operator variability (8), assist in the interpretation of findings clinicians or sonographers with limited training and experience (25), and perhaps facilitate the diffusion of point-of-care US to guide clinical decisions. Our preliminary results suggest that the integration of indexes extracted by both algorithms could provide a more reliable estimation of the volemic status than using the standard IVC assessment (26) and call for extensions of research to larger databases and other vessels, like the arteries, the evaluation of which might improve cardiovascular risk stratification (27).

In this manuscript, we discuss these methods along with some possible future applications.

## 2. Physiological Basis of Vessel Pulsatility

**Figure 1:**
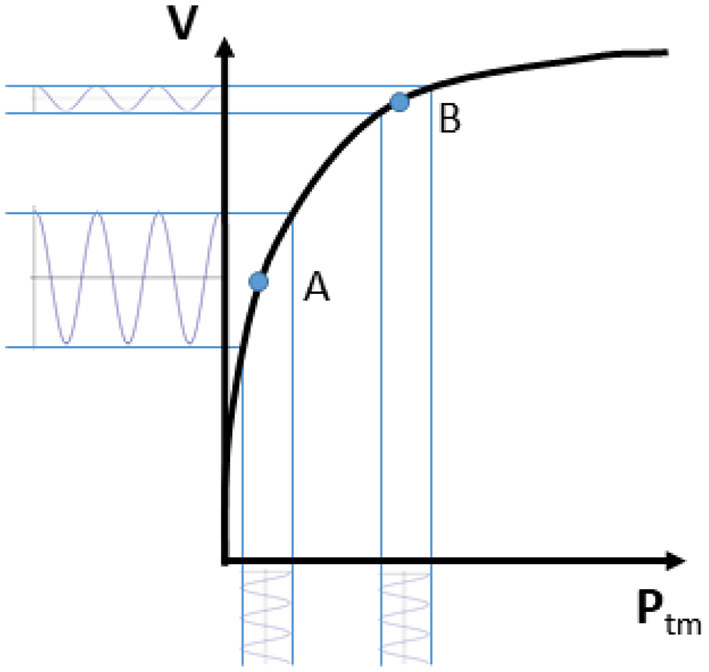
Volume-pressure curve of a venous blood vessel relating the vessel size (volume *V*) to the transmural pressure (*P*_*tm*_). Note that an oscillatory perturbation in blood pressure results in large volume changes if *P*_*tm*_ is low (A; high vessel compliance) and low pulsatility if *P*_*tm*_ is high (B; low vessel compliance).

Rapid changes in vascular size (irrespective of whether we deal with arteries or veins) are primarily produced by changes in transmural pressure *P*_*tm*_, defined as *P*_*tm*_ = *P*_*in*_−*P*_*out*_, where *P*_*in*_ is the blood pressure inside and *P*_*out*_ the pressure outside the vessel wall (28, 29). The relation between vascular size and *P*_*tm*_ is generally represented by a volume-pressure (or capacitance) curve as qualitatively shown in Figure 1, whereby the vascular size, expressed in terms of vessel volume *V*, is shown to increase with increasing transmural pressure. This sketchy representation can be assumed to be a hypothetical experimental characterization of the vessel of interest. Ranges of volume and pressure are not indicated, as they vary widely along the cardiovascular system (30). Specifically, arteries are more rigid and exposed to larger pressure variations than veins. Pressure variations in the arteries are mainly determined by the pulsatile nature of the cardiac pump and they are also affected by peripheral resistances; whilst *P*_*tm*_ changes in the veins are largely influenced by a variation of the external pressure. However, this scheme is only a simplistic representation, as intrinsic vessel characteristics, circulating blood volume, medications and many other additional factors might influence intravascular pressure (31); moreover, the volume-pressure curve of a certain vessel or vascular district can be modulated by spontaneous or drug-mediated variations in vascular tone.

Coming back to the simplified representation shown in Figure 1, it can however be observed that the curve has a tendency to flatten at high *P*_*tm*_. This indicates a decreasing vessel compliance, defined as


(1) $$\begin{array}{r} C=\frac{\Delta V}{\Delta P_{tm}} \end{array}$$

and representing the slope of the volume-pressure curve. A given perturbation of *P*_*tm*_, such as a blood pressure change of cardiac or respiratory origin, would result in a corresponding vessel volume change, according to vessel compliance. As shown in Figure 1, the same change in *P*_*tm*_ will produce different changes in vessel size, depending on the resting (average) value of *P*_*tm*_ and *V*: if average *P*_*tm*_ is low (for instance, in the case of IVC, when CVP or blood volume are normal) size changes will be large, while at a higher *P*_*tm*_ value the vessel size will be larger and its phasic changes (that we might call “pulsatility”, for simplicity) will be smaller (for instance, when CVP is high or if there is hypervolemia).

The same considerations might apply, in theory, to the entire vascular system. Notably, *P*_*tm*_ is not just dependent on the inner blood pressure, but also on the outside pressure. The effect of changes in the outside pressure are particularly relevant in veins, given their low blood pressure.

**Figure 2:**
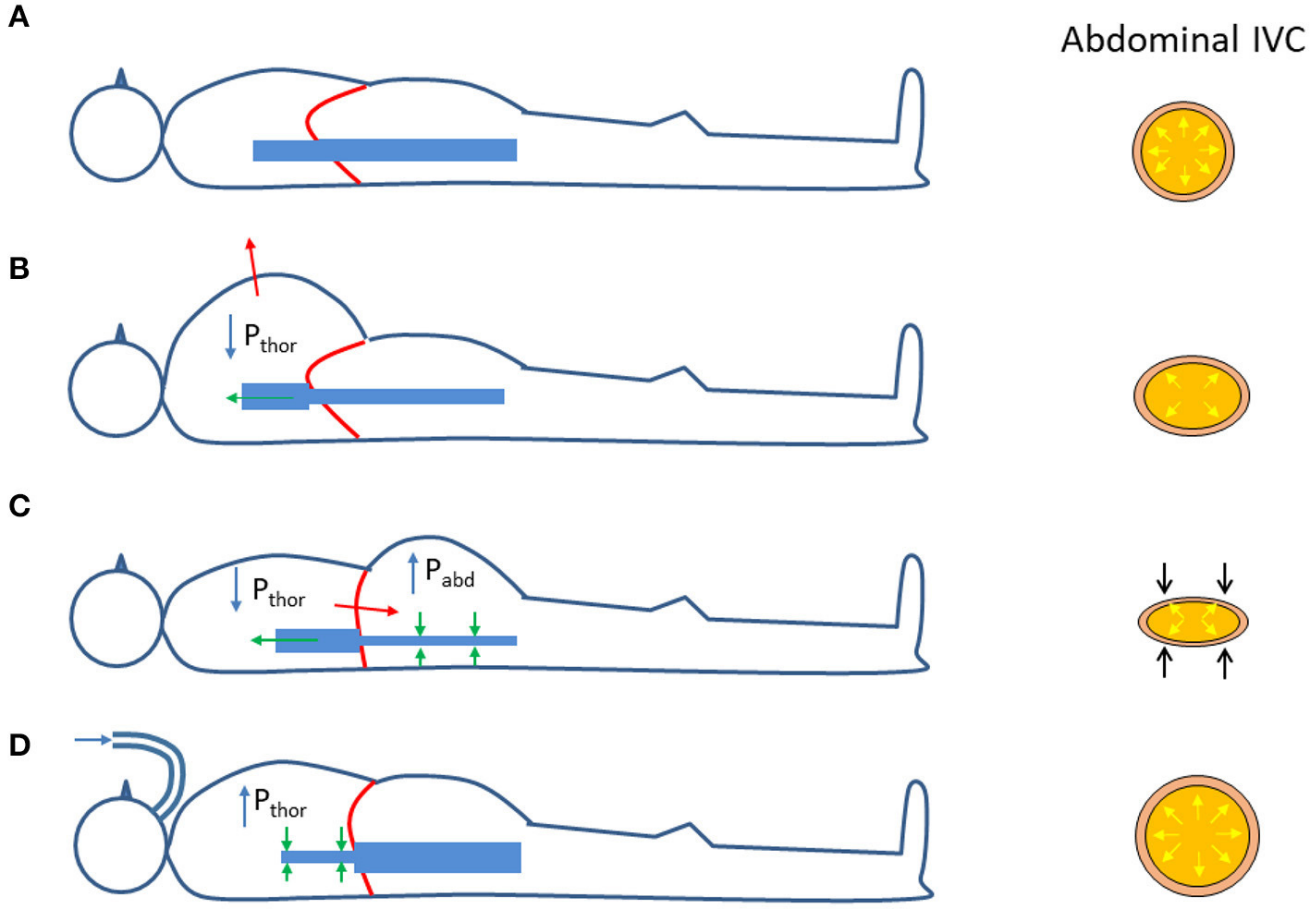
Effects of respiration on the size of the abdominal inferior vena cava (IVC); see explanation in the text. *P*_*thor*_ = Intrathoracic pressure; *P*_*abd*_ = abdominal pressure. **(A)** Functional residual capacity (end expiration). **(B)** Thoracic inspiration. **(C)** Abdominal (diaphragmatic) inspiration. **(D)** Positive pressure ventilation (inspiration).

It is worth to reconsider the changes in transmural pressure that take place in the (abdominal) IVC during respiration. As compared to the reference end-expiratory condition (Figure 2A), in which the IVC exhibits its maximum size, during a thoracic inspiration the IVC undergoes a slight reduction in size, due to the decrease in intrathoracic pressure which drains blood from the IVC, thus lowering blood pressure and its *P*_*tm*_ (Figure 2B). In addition, during inspiration, the diaphragm descends and abdominal pressure increases, with a further decrease in *P*_*tm*_ and IVC size (Figure 2C). The different implications of the respiratory pattern on IVC size have been evidenced experimentally during both spontaneous respiration (32) and in controlled isovolumetric respiratory efforts (33). Opposite effects, i.e., increased IVC size during inspiration, are observed during positive pressure ventilation, whereby the increase in intrathoracic pressure hinders venous return, thus increasing abdominal venous blood pressure and IVC size (Figure 2D).

Finally, the analysis of phasic changes of the vascular size should take in consideration an additional confounding factor: the extravascular compliance. Since blood vessels are embedded within other organs and tissues, their possibility to expand upon variations in blood pressure depends on the capacity of the extravascular tissues to accommodate such changes. In other words, when measuring vessel volume changes in response to given variations in blood pressure, we are actually assessing the total compliance (*C*_*tot*_), which accounts for the vascular (*C*_*v*_) and extravascular (*C*_*ev*_) compliances according to the formula


(2) $$\begin{array}{r} C_{tot}=\frac{1}{\frac{1}{C_{v}}+\frac{1}{C_{ev}}} \end{array}$$

*C*_*tot*_ resulting smaller than *C*_*v*_.

In summary, 1) the vessel size depends on *P*_*tm*_ = *P*_*in*_−*P*_*out*_, according to a non-linear volume-pressure curve; 2) the vessel compliance (*C* = Δ*V*/Δ*P*_*tm*_) generally decreases at increasing *P*_*tm*_; 3) vessel phasic changes depend on vascular compliance; 4) low extravascular compliance may lead to underestimate actual vessel compliance.

## 3. Echography Processing

**Figure 3:**
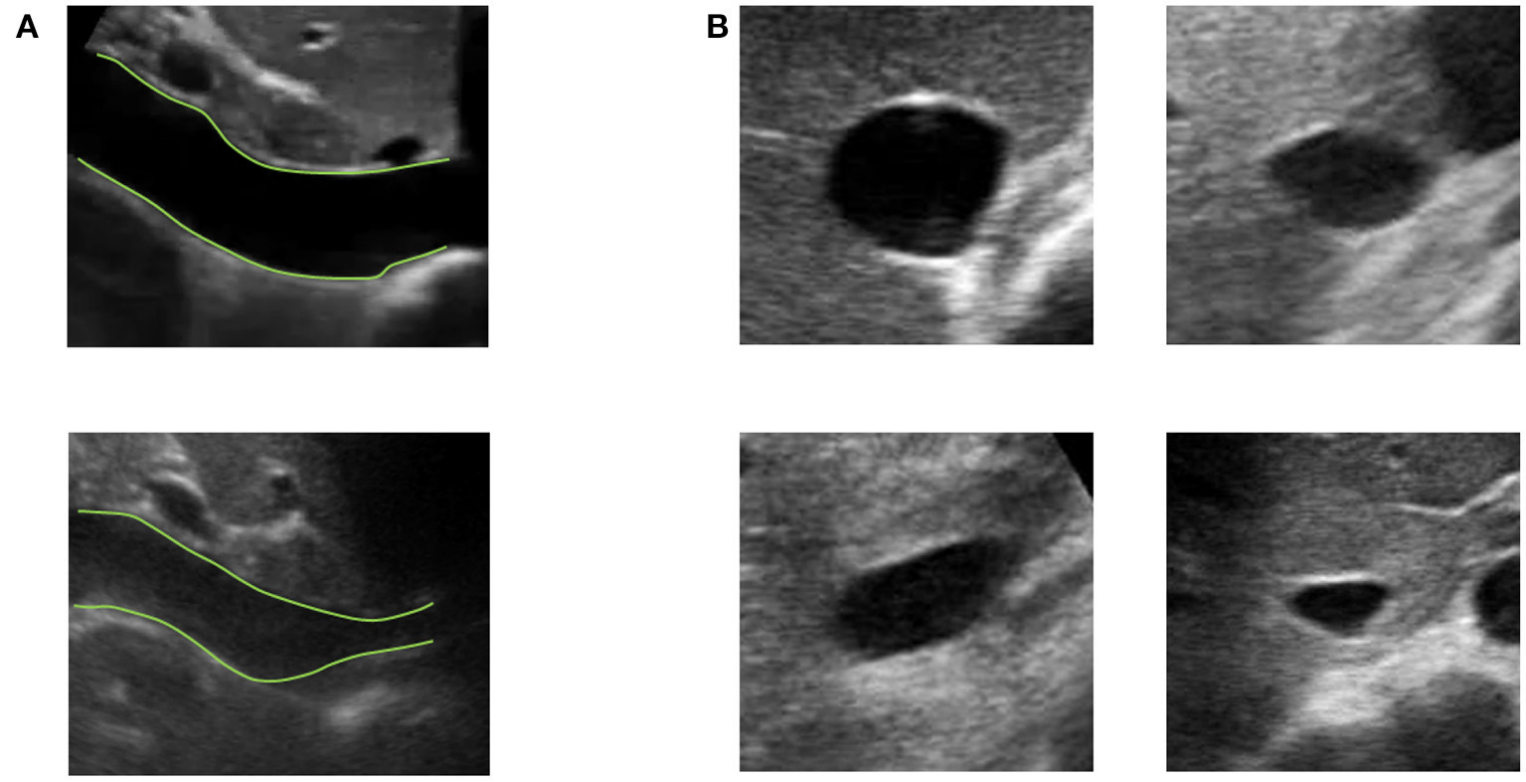
Examples of long and short axis sections of inferior vena cava (IVC) from different subjects. **(A)** IVC in long axis: one IVC has a stable diameter whereas the other shows great variations along the longitudinal axis. **(B)** Different shapes of IVC cross-sections.

**Figure 4:**
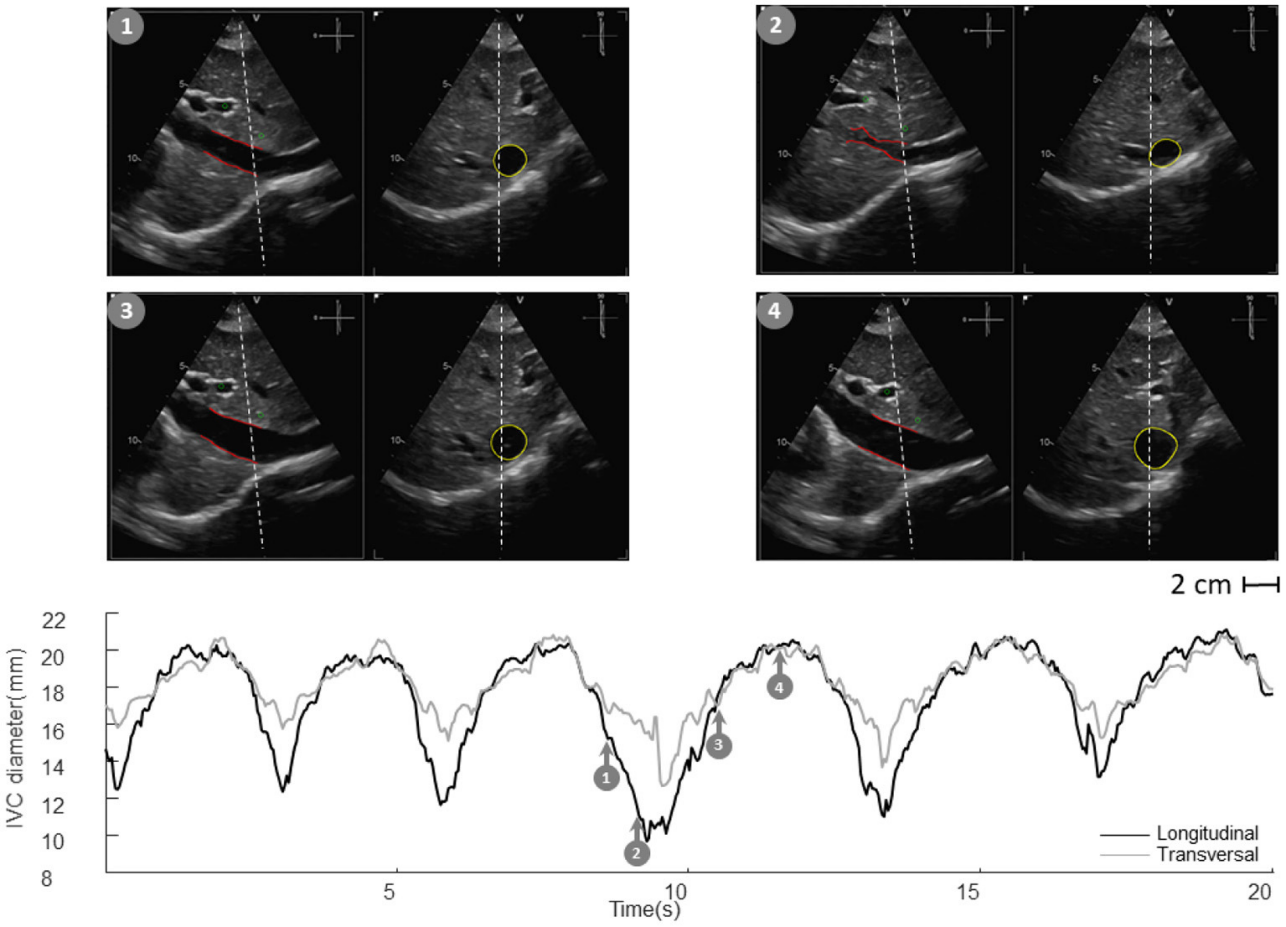
Inferior vena cava (IVC) echography in x-plane. Both long and short axis views are available in synchronous scans. The sections can be displaced with respect to the center of the vessel. Moreover, they could be not orthogonal to the IVC axis. The estimation of the diameter in a single section can be largely affected.

Vessels such as the IVC might have a complex geometry. In particular, as shown in Figure 3, the section of the IVC is not constant along the longitudinal axis and its cross-section is far from being like a perfect circle, with large variations across subjects and clinical conditions. Moreover, IVC is a very compliant vessel whose movements are also affected by surrounding structures to which it can be anchored. Therefore, measuring its size on a single plane might be largely inaccurate (as shown in Figure 4).

US scans of IVC in B-mode provide limited information on its phasic changes, which occur in a three dimensional space. With our two algorithms we can estimate the IVC edges either in long or short axis views (12, 23).

In long axis, IVC movements are estimated by tracking two reference points selected by the user. Then, the edges are estimated in an entire longitudinal portion of the vessel. The diameters of different sections are then computed in directions orthogonal to the midline of the IVC, thus compensating for possible translations and rotations in the visualized plane (23).

In the case of the short axis view, the contour of the cross-section of the IVC is estimated, finding the edges along different directions starting from its center, identified in the previous frame as the centroid of the vessel border (12).

As the edges of the IVC are estimated in each frame of the US video, a temporal series is acquired, from which different measures of IVC size can be obtained (e.g., different diameters, their average, or the cross-sectional area). In particular, two main contributions are clearly visible in IVC dynamics described by those time series: a slow oscillation of IVC size induced by respiration (due to variations of intrathoracic and abdominal pressure) and another at higher frequency induced by the heartbeats (reflecting retrograde flow induced by right atrial contraction). These two components can be measured separately (respiratory caval index, RCI, and cardiac caval index, CCI) and potentially provide complementary information (4, 8, 12, 23, 24, 26, 34).

Alternative methods to assess phasic changes of vascular size have also been applied or developed by other colleagues (35–37). Specifically, a method widely used in echocardiography is based on tracking the speckle noise (38), i.e., a random mixture of interference patterns and US reflections characterizing each region of the tissue (like as a fingerprint) that is relatively stable on consecutive frames, allowing the region to be traced from one frame to the next. Speckle tracking has been applied to study the IVC deformation in long axis view (36) or to assess aortic or carotid stiffness (39, 40). Different processing techniques have also been used to segment blood vessels: for example, Otsu's thresholding (41) was combined with active contour on multiple short axis views to estimate the carotid in 3D (42); a semi-automated modified watershed method was applied to estimate the cross-section of IVC (43); snake and template matching (the latter approach, similar to speckle tracking) were tested for IVC edge detection in short axis (44); a novel energy functional for polar active contour was applied to the segmentation of IVC in short axis (45). More recently, deep learning approaches have been applied on the segmentation of IVC in short axis, obtaining moderately good performances in predicting fluid responsiveness (46). Edge-tracking methodology might exhibit a good compromise between computational cost and accuracy (12). We are currently working on the real time implementation and rendering over the US scan of the estimated vascular edges: this can provide the operators with visual feedback and guidance to obtain and acquire good quality images; moreover, the simultaneous measurement of quantitative indexes (e.g., mean diameter and pulsatility indexes) might be a valuable add-on for research and routine clinical practice.

## 4. Current and Future Applications

**Figure 5:**
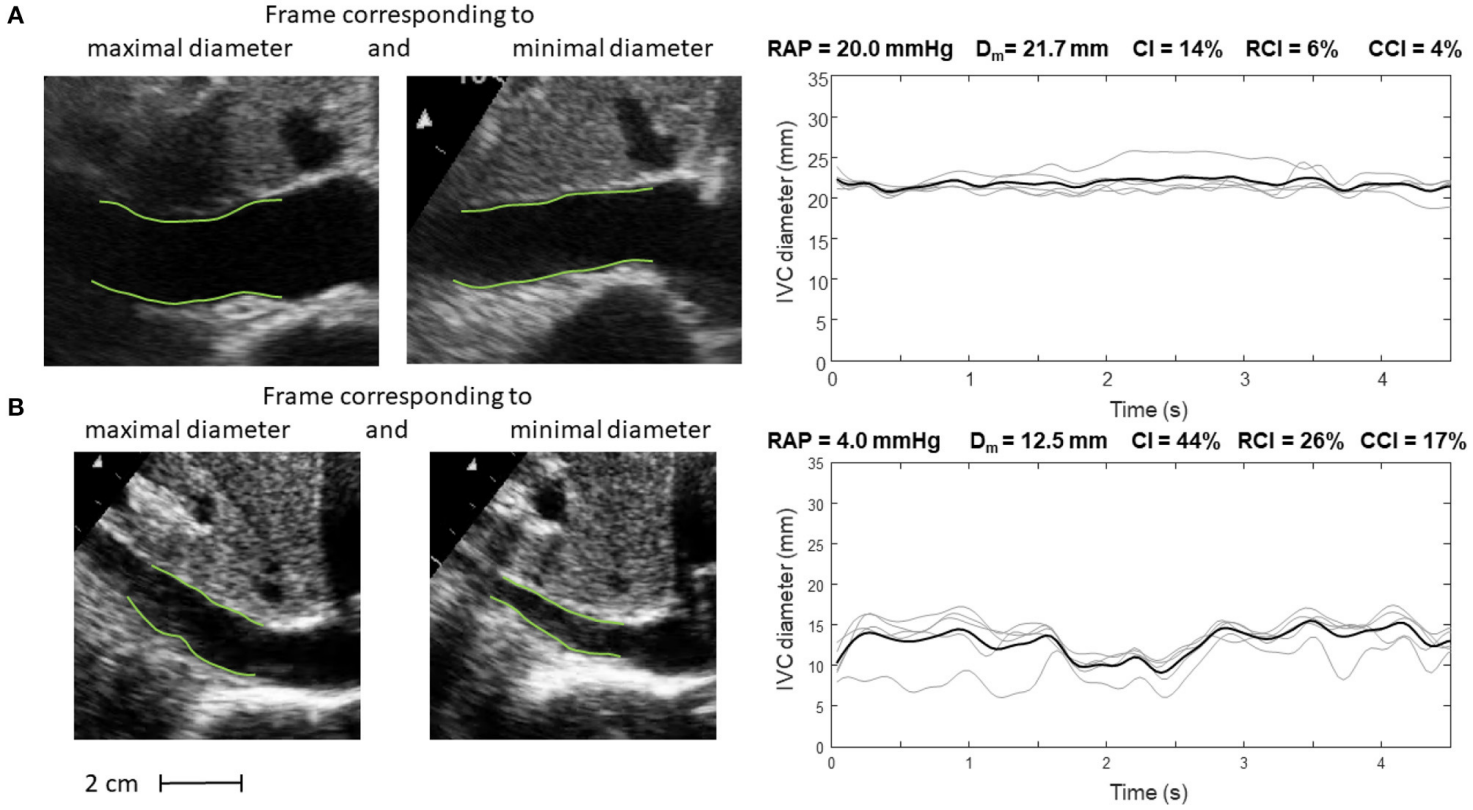
Inferior vena cava (IVC) dynamics in patients with different right atrial pressure (RAP, measured invasively). **(A)** Patient with high RAP. **(B)** Patient with low RAP. Abbreviations used: average diameter *D*_*m*_, caval index *CI*, respiratory caval index *RCI*, cardiac caval index *CCI*.

**Figure 6:**
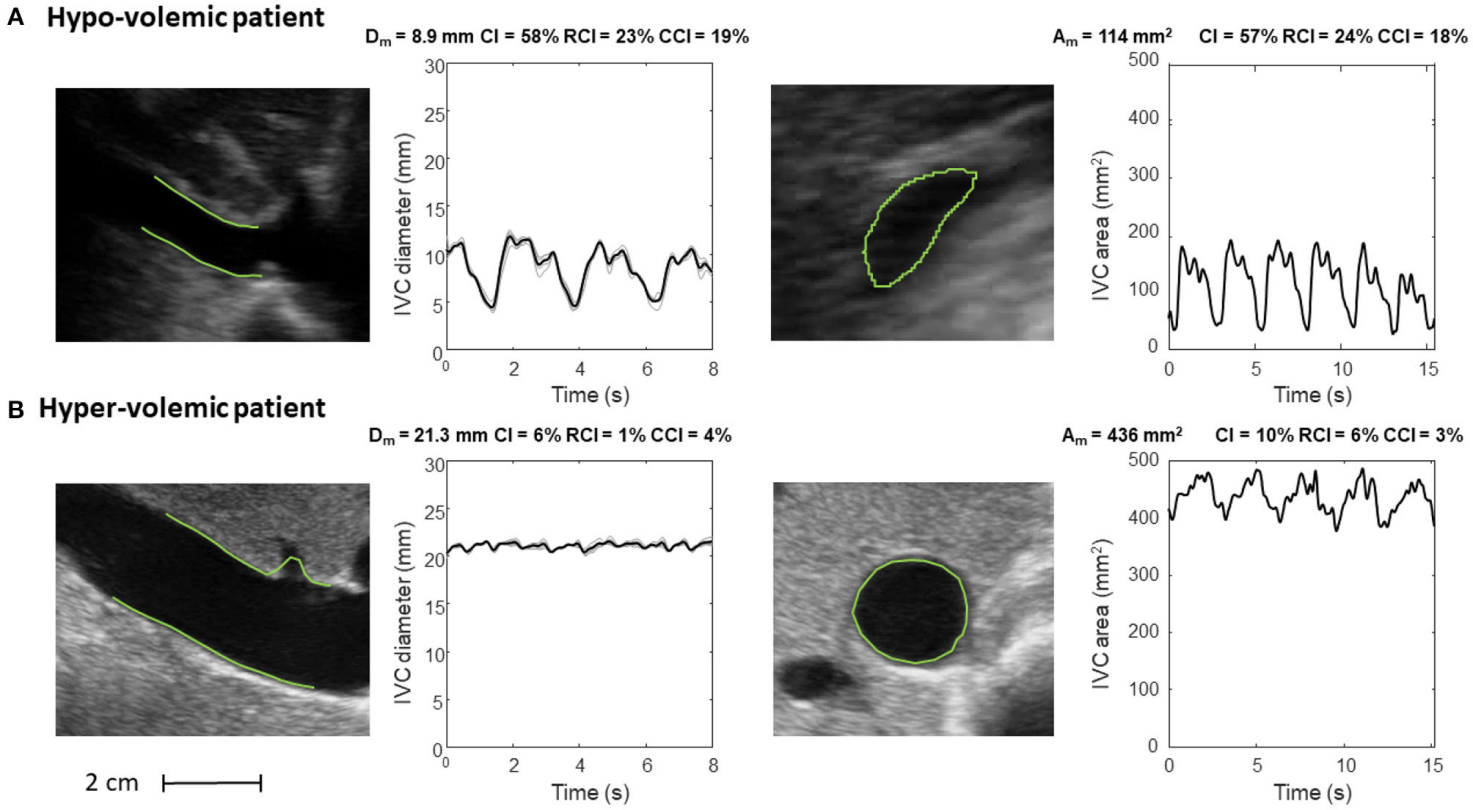
Inferior vena cava (IVC) dynamics in patients with different volume status. **(A)** Hypo-volemic patient. **(B)** Hyper-volemic patient. Abbreviations used: average diameter *D*_*m*_, average cross-sectional area *A*_*m*_, caval index *CI*, respiratory caval index *RCI*, cardiac caval index *CCI*.

Our methods have been applied in two pilot studies, to estimate the RAP (24, 34) and volume status (26). Representative examples are shown in Figures 5, 6.

Specifically, Figure 5 shows frames corresponding to maximal and minimal IVC size for two patients, with invasively measured high (A) and low (B) RAP, respectively. The average size of IVC is larger in the patient with higher RAP (20 mmHg) and the IVC phasic changes are greater for the patient with lower RAP (4 mmHg): in the latter case, small variations of transmural pressure induce large changes of size in the vessel.

Figure 6 shows the long and short axis of the IVC in a hypo- (Figure 6A) and hyper- (Figure 6B) volemic patient, respectively. Compared to patients with hypervolemia, in those with hypovolemia the IVC size is smaller and the pulsatility is larger; moreover, the IVC in the cross-section view has a flattened shape, whereas it is mostly circular in conditions of fluid overload.

In addition, the features offered by the automated algorithms (i.e., size and indexes of pulsatility of the blood vessel of interest) open the way to new applications. We here briefly overview those that are of particular interest to the authors, i.e., specific studies or applications that have recently been attempted and are close to completion or that belong to the author's different working fields in basic and clinical sciences and that will be addressed in the near future. These existing and potential application of the methodology are presented in Table 1, but many other applications may possibly be envisaged.

**Table 1:** Current evaluation and potential clinical relevance of image-processing algorithms applied to vascular ultrasound.

**Current evaluation**	**Potential clinical relevance**
Tracking of IVC movements and	Assessment of volemic status
phasic deformation, longitudinally	Estimation of right atrial pressure
or cross-sectionally	Assessment of venous compliance and fluid redistribution
Tracking of peripheral veins deformation	Monitoring responses during renal dialysis, to diuretics or a fluid challenge
Tracking of arterial phasic deformation, longitudinally or	Evaluation of arterial stiffness
cross-sectionally	Assessment of cardiovascular health

Edge tracking algorithms can be applied to peripheral veins to investigate, by ultrasound, the mechanical response to changes in transmural pressure, e.g., by venous occlusion, for the assessment of venous compliance (47, 48) and characterize the filling condition and the expanding capacity of the peripheral reservoir, a major pathway for venous return. Assessment of venous compliance could also be used to validate another recently proposed index of peripheral vascular filling, the venous pulse wave velocity (49, 50). Combining these methods with the IVC assessment might increase the understanding of the underlying mechanisms of fluid distribution and displacements across different body regions and compartments in various clinical contexts (28, 29).

In this respect, an interesting model of acute fluid redistribution is offered by the MuVIT technique (51), a procedure used to transiently lower aortic blood flow and pressure during thoracic or abdominal vascular interventions (e.g., stent graft placements) consisting in transiently increasing alveolar pressure up to about 30 mmHg. This maneuver provokes a substantial blood volume displacement from the pulmonary and arterial compartments to the systemic venous compartment resulting in a transient venous congestion. The possibility of simultaneous tracking size changes of abdominal and peripheral veins may help to characterize the dynamic behavior of the full venous compartment.

Fluid overload, or congestion, is a key clinical feature in acute heart failure, but its management with diuretic is still very subjective (52). Controversial results have been documented in the literature about the potential utility of IVC diameter and distensibility to monitor the response to diuretics in patients with acute heart failure (53, 54). Quantifying with precision phasic IVC changes might potentially detect even small variations in intravascular fluids and possibly guide clinicians for a more objective use of diuretic therapy.

Similar considerations apply to renal failure patients undergoing dialysis, whereby automated continuous and unsupervised IVC monitoring may help to tailor the dialytic process according to the current volume status of the patient.

The possibility to detect a cardiac pulsatility of IVC in addition to the major oscillatory component of respiratory origin is still largely unexplored and many questions have to be addressed. May the cardiac pulsatility provide a more reliable index of IVC collapsibility than the classical caval index? Is cardiac pulsatility carrying additional or different information than respiratory phasic variations? Long term monitoring and correlation analysis of these oscillatory components as well as extending the investigation to specific patient groups is necessary to address these questions.

Finally, a relevant application concerns the assessment of arterial stiffness. Aortic stiffness is associated with incident cardiovascular events. In clinical practice, aortic stiffness is usually investigated indirectly in terms of the PWV. However, the PWV is usually measured from the carotid-femoral artery PWV, a global index, which does not reflect local stiffness variations. In theory, aortic stiffness could be estimated directly, by measuring with US the aorta pulsatility under a known pressure variation (systo-diastolic) (55), in different aortic segments (56). Whether segmental aortic stiffness measured by US might discriminate different patients' conditions and risk, it is worth exploring.

## 5. Conclusions

Rapid advances in US image processing have made now possible to obtain more objective information on the size of arteries and veins, but also to quantify their phasic changes with more precision, which can transform management of several conditions and potentially improve outcomes.

## 6. Patents

An instrument implementing the algorithms for IVC delineation used in this paper was patented by Politecnico di Torino and Universitá di Torino (WO 2018/134726).

## Author Contributions

LM, SA, and SR: methodology and writing–original draft preparation. LM and PPo: software. SA, SR, PPa, MP, and LE: data preparation. LM, SR, and PPo: visualization. MP, GS, PPe, FA-C, and SR: supervision. All authors conceptualization, writing–review, editing, read, and agreed to the published version of the manuscript.

## Funding

Supported by the Italian Ministry of Economic Development through the Proof of Concept (PoC-Off) project Vein Image Processing for Edge Rendering–VIPER (CUP C16I20000080006).

## Conflict of Interest

RB was employed by company Chirurgia Generale e Trauma Team GOM Niguarda. The remaining authors declare that the research was conducted in the absence of any commercial or financial relationships that could be construed as a potential conflict of interest.

## Publisher's Note

All claims expressed in this article are solely those of the authors and do not necessarily represent those of their affiliated organizations, or those of the publisher, the editors and the reviewers. Any product that may be evaluated in this article, or claim that may be made by its manufacturer, is not guaranteed or endorsed by the publisher.
